# Supplementary material for: In Silico Identification and Characterization of circRNAs as Potential Virulence-Related miRNA/siRNA Sponges from Entamoeba histolytica and Encystment-Related circRNAs from Entamoeba invadens
Source: Noncoding RNA. 2022 Sep 26;8(5):65. doi: 10.3390/ncrna8050065 (PMC9607107; doi:10.3390/ncrna8050065)
Supplement: Supplementary file 1 [file ncrna-08-00065-s001.zip › ncrna-1896076-supplementary.pdf]

Supplementary Material

1. Supplementary Figures

Figure S1. Workflow for identification and partial characterization of circRNAs from *Entamoeba histolytica* and *Entamoeba invadens*. The programs used in each step are depicted in small letters.

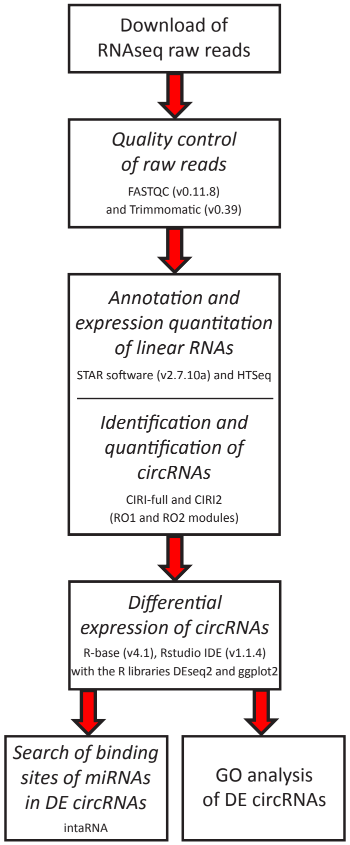

Figure S2. Design and rescue of synthetic circRNA decoy sequence from limited *Entamoeba histolytica* total RNA-Seq libraries

Decoy sequence used:

ATTAAGGTTGCATCTAAGACTGCTAAGAAAGCTGCTAAAGCAGAAAAGAAGGCTGAAAGCAAATTAAGCAAGATTAAGAAGGATGTTAAGAAAGGAACTAAGACTGCTGCTCAACTCAAGAAGGTACAATCTCAA

Rescued sequences

Without Decoy

circRNA_ID	chr	circRNA_start	circRNA_end	#junction_reads	SM_MS_SMS	#non_junction _reads	junction_reads _ratio	circRNA_type	gene_id	strand	junction_reads_ID
DS571145:504073|504213	DS571145	504073	504213	1	1_1_0	10	0.167	exon	EHI_153780	-	ERR058005.11348,
DS571153:94757|94905	DS571153	94757	94905	3	3_1_0	3697	0.002	exon	EHI_023230	-	ERR058005.441055,ERR058005.567414,ERR058005.626926,
DS571163:34232|34375	DS571163	34232	34375	2	2_2_0	161	0.024	exon	EHI_096350	+	ERR058005.65878,ERR058005.1871775,
DS571168:92541|92882	DS571168	92541	92882	1	1_0_1	236	0.008	exon	EHI_013220	+	ERR058005.80069,
DS571191:16879|17058	DS571191	16879	17058	1	0_1_1	89	0.022	exon	EHI_194220	+	ERR058005.1571799,
DS571234:36366|37151	DS571234	36366	37151	1	1_1_0	66	0.029	exon	EHI_059870	+	ERR058005.1410681,
DS571244:24189|24328	DS571244	24189	24328	2	2_0_1	1578	0.003	exon	EHI_146110	+	ERR058005.346566,ERR058005.1214370,
DS571253:31443|31610	DS571253	31443	31610	1	1_1_0	15	0.118	exon	EHI_030420	+	ERR058005.396614,
DS571292:16575|17213	DS571292	16575	17213	1	1_1_0	624	0.003	intergenic_region	n/a	+	ERR058005.317030
DS571377:17778|18245	DS571377	17778	18245	2	1_2_0	2	0.667	exon	EHI_169670	-	ERR058005.1468940,ERR058005.816067,

With Decoy

circRNA_ID	chr	circRNA_start	circRNA_end	#junction_reads	SM_MS_SMS	#non_junction_	junction_reads_	circRNA_type	gene_id	strand	junction_reads_ID
DS571145:504073|504213	DS571145	504073	504213	1	1_1_0	11	0.154	exon	EHI_153780,	-	ERR058005.11348,
DS571151:141804|141938	DS571151	141804	141938	10	1_9_0	493	0.039	exon	EHI_110740	+	ERR058005.xxxxxx1,ERR058005.xxxxxx2,ERR058005.xxxxxx3,ERR058005.xxxxxx4,ERR058005.xxxxxx5,ERR058005.xxxxxx6,ERR058005.xxxxxx7,ERR058005.xxxxxx8,ERR058005.xxxxxx9,ERR058005.xxxxx10,
DS571153:94757|94905	DS571153	94757	94905	3	3_1_0	3697	0.002	exon	EHI_023230,	-	ERR058005.441055,ERR058005.567414,ERR058005.626926,
DS571163:34232|34375	DS571163	34232	34375	2	2_2_0	161	0.024	exon	EHI_096350,	+	ERR058005.65878,ERR058005.1871775,
DS571168:92541|92882	DS571168	92541	92882	1	1_0_1	236	0.008	exon	EHI_013220,	+	ERR058005.80069,
DS571191:16879|17058	DS571191	16879	17058	1	0_1_1	89	0.022	exon	EHI_194220,	+	ERR058005.1571799,
DS571234:36366|37151	DS571234	36366	37151	1	1_1_0	66	0.029	exon	EHI_059870,	+	ERR058005.1410681,
DS571244:24189|24328	DS571244	24189	24328	2	2_0_1	1578	0.003	exon	EHI_146110,	+	ERR058005.346566,ERR058005.1214370,
DS571253:31443|31610	DS571253	31443	31610	1	1_1_0	15	0.118	exon	EHI_030420,	+	ERR058005.396614,
DS571292:16575|17213	DS571292	16575	17213	1	1_1_0	624	0.003	intergenic_region	n/a	+	ERR058005.317030,
DS571377:17778|18245	DS571377	17778	18245	2	1_2_0	2	0.667	exon	EHI_169670,	-	ERR058005.1468940,ERR058005.816067,

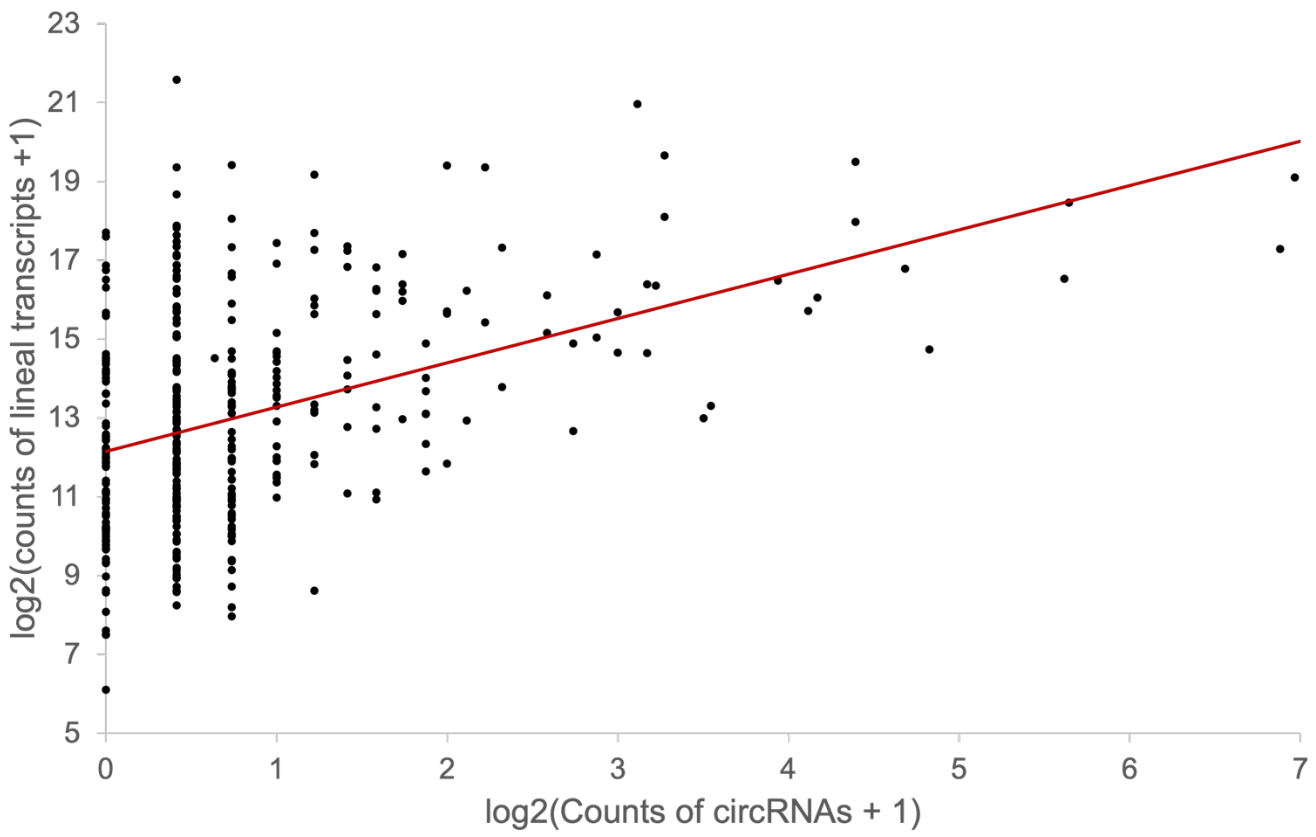

Figure S3. Linear correlation between circRNA and mRNA expression in *Entamoeba histolytica*.

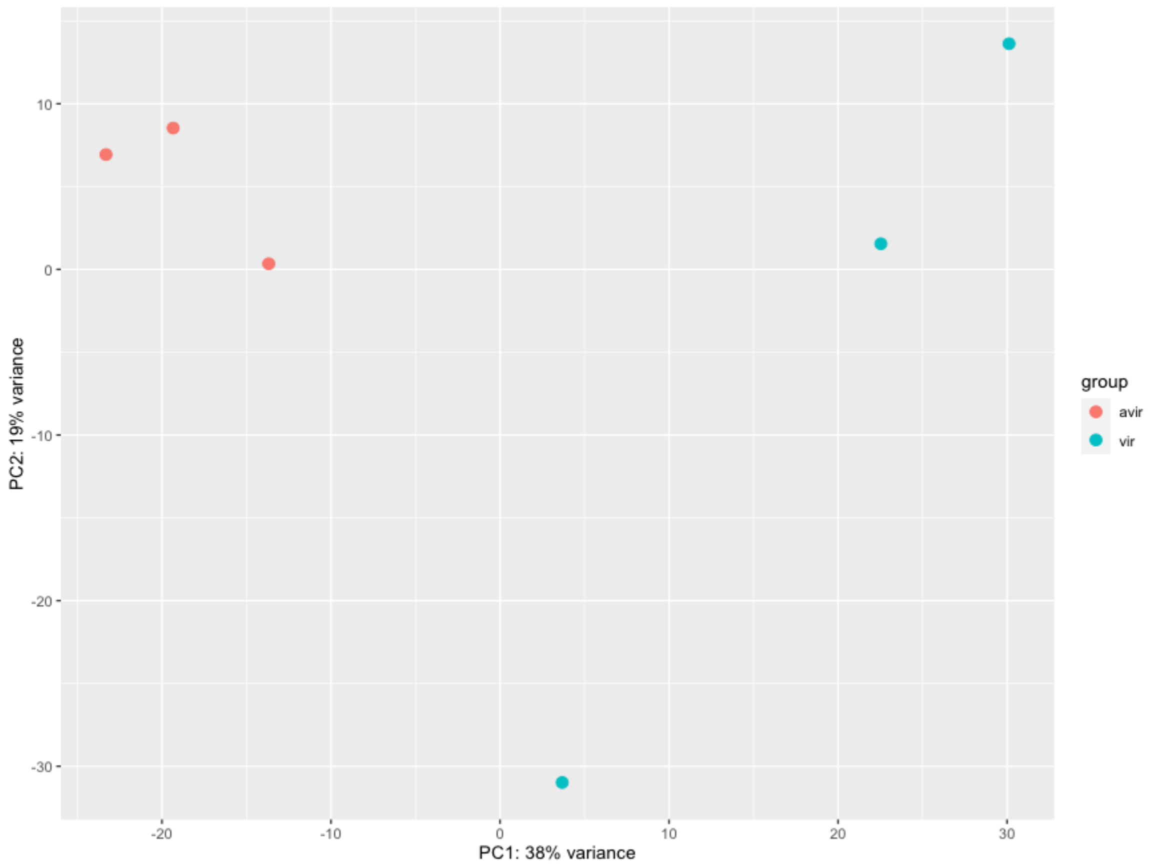

Figure S4. Principal Components Analysis of the six total RNA-Seq libraries used in this work. The virulent *Entamoeba histolytica* HM-1:IMSS libraries are plotted in blue dots. The avirulent Rahman E. histolytica libraries are plotted in red dots.

2. Supplementary Tables

Table S1. Metabolic process GO terms of *Entamoeba histolytica* HM1:IMSS strain circRNAs

	term_ID	description	frequency	plot_X	plot_Y	log_size	Log_pvalue	uniqueness	dispensability
1	GO:0006414	translational elongation	0.493289439	7.65094583	0.215479206	5.077077067	-3.38019626	0.63096898	0
2	GO:0032270	positive regulation of cellular protein metabolic process	0.436301681	-2.639331026	-6.142893568	5.02376261	-1.352638978	0.973797674	0
3	GO:0097435	supramolecular fiber organization	0.320921711	-4.651720492	1.525521652	4.890376301	-1.974348337	0.922195548	0.00940424
4	GO:0030029	actin filament-based process	0.379280877	-3.777717299	-4.412659159	4.962937288	-1.506154129	0.983540201	0.00954858
5	GO:0046034	ATP metabolic process	1.401615485	-4.670852677	-2.619475694	5.530601779	-2.7441943	0.922868068	0.03618763
6	GO:0009058	biosynthetic process	24.55552771	-0.031193729	6.812710734	6.774120957	-5.153380191	0.872684796	0.06111925
7	GO:0016052	carbohydrate catabolic process	1.344206391	-1.76364047	-1.493405454	5.512438902	-2.421387171	0.834639186	0.07108273
8	GO:0019693	ribose phosphate metabolic process	2.92249384	1.015326329	-5.675694941	5.84972583	-2.761330343	0.550617254	0.0723429
9	GO:0032787	monocarboxylic acid metabolic process	2.785819879	4.577313194	-6.547867677	5.828925289	-1.322687681	0.660513081	0.08994923
10	GO:0006091	generation of precursor metabolites and energy	3.310573204	-2.674585496	3.722490095	5.903875349	-2.7441943	0.860267836	0.09218577
11	GO:0044281	small molecule metabolic process	15.96586237	-2.159924017	5.825774706	6.587164106	-1.506362434	0.884317582	0.10702689
12	GO:0034641	cellular nitrogen compound metabolic process	26.56565976	2.593669342	1.046492403	6.808292286	-4.782213837	0.748213555	0.13517013
13	GO:0044238	primary metabolic process	50.27438061	1.174698985	5.588388044	7.085318376	-1.343116896	0.846965067	0.17218878
14	GO:0010467	gene expression	11.65274909	6.532144496	5.334749692	6.450400161	-4.777472704	0.80964225	0.18543611
15	GO:0072521	purine-containing compound metabolic process	2.690932536	6.420385601	-2.195366118	5.813875088	-2.551540769	0.668128346	0.21807938
16	GO:0006807	nitrogen compound metabolic process	45.78921766	1.821822306	6.420678001	7.044734875	-1.334784784	0.8508187	0.23601289
17	GO:0043603	cellular amide metabolic process	7.027632371	7.079078064	-2.229850435	6.230780902	-8.168546862	0.671290565	0.3104676
18	GO:1901564	organonitrogen compound metabolic process	32.51688194	3.246522142	2.116541702	6.896080561	-2.419007832	0.744114702	0.34582176
19	GO:0044267	cellular protein metabolic process	15.2980696	5.613712904	2.030517962	6.568608362	-1.418864815	0.676951198	0.35919996
20	GO:0046939	nucleotide phosphorylation	0.594843806	0.620659349	-6.622809086	5.158377572	-2.159425662	0.704452119	0.38991675
21	GO:0007015	actin filament organization	0.206124176	-4.739473403	0.734601151	4.698109249	-2.150714175	0.875295571	0.40084008
22	GO:0044271	cellular nitrogen compound biosynthetic process	14.24186289	5.779822816	-0.66026011	6.537538538	-8.035161754	0.562849582	0.42282582
23	GO:0019538	protein metabolic process	19.93516961	4.967132475	2.414507328	6.683591637	-1.528377661	0.694785225	0.43443254
24	GO:0055086	nucleobase-containing small molecule metabolic process	5.183027529	5.88184902	-3.428913942	6.098555474	-2.212267747	0.58326204	0.47146561
25	GO:0009059	macromolecule biosynthetic process	11.33841591	7.22098644	2.999571739	6.438524154	-6.221752614	0.694724366	0.51353713
26	GO:0006413	translational initiation	0.515768954	7.503807702	-0.009312863	5.096430287	-2.152584301	0.629326829	0.52406218
27	GO:0007010	cytoskeleton organization	0.818651702	-4.698161545	1.079430522	5.297072972	-1.468700619	0.903593626	0.54693131
28	GO:1901566	organonitrogen compound biosynthetic process	15.3359568	5.9858644	0.843195022	6.569682605	-7.213720399	0.627657137	0.55409463
29	GO:0044264	cellular polysaccharide metabolic process	0.720257355	5.247584244	5.189734113	5.241461805	-1.352638978	0.78780213	0.57375567
30	GO:0009206	purine ribonucleoside triphosphate biosynthetic process	0.491170367	4.945518533	-3.659909631	5.075207422	-1.710063737	0.419944316	0.57519551
31	GO:0006096	glycolytic process	0.532804146	3.627267988	-3.713939785	5.110542575	-2.159425662	0.401943532	0.58053335
32	GO:0009132	nucleoside diphosphate metabolic process	0.690020302	4.179412457	-4.742280048	5.222836081	-2.060038045	0.500051771	0.59968967
33	GO:0044249	cellular biosynthetic process	22.87469451	7.325857975	1.287619466	6.743326993	-6.083290757	0.664367507	0.63246679
34	GO:0006412	translation	5.346943753	6.600053193	0.308538983	6.112077563	-8.798949261	0.516909689	0.67137328
35	GO:0006090	pyruvate metabolic process	0.818337765	4.208488186	-7.087767081	5.296906398	-1.882374145	0.695110835	0.69337288

Table S2. Metabolic process GO terms of *Entamoeba histolytica* Rahman strain circRNAs

	term_ID	description	frequency	plot_X	plot_Y	log_size	Log_pvalue	uniqueness	dispensability
1	GO:0006414	translational elongation	0.493289439	-6.029494701	3.093196489	5.077077067	-3.132943016	0.751575959	0
2	GO:0008152	metabolic process	62.77472966	4.292832107	-4.835193606	7.181756492	-1.694018247	1	0
3	GO:0043044	ATP-dependent chromatin remodeling	0.058309597	3.066843647	-3.497200063	4.149742415	-1.502185499	0.956297521	0.00814723
4	GO:0055082	cellular chemical homeostasis	0.412744042	5.343504558	-1.362747265	4.999656772	-2.247236514	0.790069115	0.00962332
5	GO:0046034	ATP metabolic process	1.401615485	2.469843223	-5.7928734	5.530601779	-2.876650801	0.949920703	0.03618763
6	GO:0009058	biosynthetic process	24.55552771	0.997464326	-2.008068133	6.774120957	-3.328405302	0.917658442	0.06111925
7	GO:0006091	generation of precursor metabolites and energy	3.310573204	0.409365599	-4.493665696	5.903875349	-2.876650801	0.906401951	0.07337732
8	GO:0009056	catabolic process	6.023915031	0.686937655	-6.503181027	6.163850747	-1.467955564	0.93761913	0.08226917
9	GO:0005975	carbohydrate metabolic process	5.962693259	0.136824525	8.812833906	6.159414381	-2.936840498	0.90425223	0.08410537
10	GO:0034641	cellular nitrogen compound metabolic process	26.56565976	-2.039806417	3.298619239	6.808292286	-2.361133582	0.832340768	0.13517013
11	GO:0044238	primary metabolic process	50.27438061	-1.342408704	8.075322806	7.085318376	-1.526933972	0.901593148	0.17218878
12	GO:0006096	glycolytic process	0.532804146	-4.579928706	-1.870536153	5.110542575	-2.894651312	0.610424265	0.18459208
13	GO:0010467	gene expression	11.65274909	-3.850079958	7.379784143	6.450400161	-2.497415645	0.871484852	0.18543611
14	GO:0007264	small GTPase mediated signal transduction	0.257304107	5.217200468	2.350373901	4.794425303	-1.665360792	0.78209225	0.196147
15	GO:0043618	regulation of transcription from RNA polymerase II promoter in response to stress	0.019092303	4.787461299	2.759044585	3.664923893	-1.502185499	0.73159501	0.26690368
16	GO:0072521	purine-containing compound metabolic process	2.690932536	-4.184571267	1.050762493	5.813875088	-2.154965188	0.784137774	0.26793713
17	GO:0031328	positive regulation of cellular biosynthetic process	0.837223531	5.879225189	-0.844012821	5.306815181	-1.426057453	0.848299182	0.28544421
18	GO:0042780	tRNA 3'-end processing	0.049527633	-7.086780791	0.708029547	4.078855403	-1.502185499	0.812313092	0.2939335
19	GO:1901700	response to oxygen-containing compound	0.554783841	4.214040046	5.109980302	5.128098648	-1.502185499	0.76142678	0.31224321
20	GO:1901564	organonitrogen compound metabolic process	32.51688194	-2.47672999	5.454424495	6.896080561	-2.324413465	0.833432877	0.34582176
21	GO:0044271	cellular nitrogen compound biosynthetic process	14.24186289	-4.643875581	2.469481264	6.537538538	-5.606035384	0.712833335	0.34987879
22	GO:0019538	protein metabolic process	19.93516961	-3.581411072	4.814376522	6.683591637	-1.83018621	0.795521412	0.3677588
23	GO:0043620	regulation of DNA-templated transcription in response to stress	0.020063029	5.154268622	2.875544104	3.68645751	-1.502185499	0.761871075	0.39242859
24	GO:0055086	nucleobase-containing small molecule metabolic process	5.183027529	-5.314488896	-0.123375912	6.098555474	-1.908763498	0.731583669	0.39660054
25	GO:0043603	cellular amide metabolic process	7.027632371	-4.685388863	1.041070885	6.230780902	-5.539544064	0.7853298	0.42282582
26	GO:0006979	response to oxidative stress	0.582480486	4.898734578	3.942916766	5.149256072	-1.502185499	0.792548207	0.4264875
27	GO:0044267	cellular protein metabolic process	15.2980696	-4.13323287	4.045342451	6.568608362	-1.639096964	0.775538585	0.43443254
28	GO:0046939	nucleotide phosphorylation	0.594843806	-3.273812241	-5.503496686	5.158377572	-2.894651312	0.832641791	0.45344761
29	GO:0006427	histidyl-tRNA aminoacylation	0.041005906	-6.300346265	0.747096309	3.996861768	-1.502185499	0.753388071	0.45717927
30	GO:0006415	translational termination	0.184136221	-5.355193643	2.268752669	4.64912058	-1.502185499	0.744123423	0.47756623
31	GO:0032787	monocarboxylic acid metabolic process	2.785819879	-5.786119257	-3.315875584	5.828925289	-1.446593115	0.794942545	0.49534084
32	GO:0051125	regulation of actin nucleation	0.023813745	4.434263688	-1.655019255	3.760874638	-1.502185499	0.820072258	0.49965912
33	GO:0009059	macromolecule biosynthetic process	11.33841591	-5.16011174	5.765267642	6.438524154	-4.12911937	0.806437913	0.51353713
34	GO:0006413	translational initiation	0.515768954	-6.051011397	2.873395092	5.096430287	-1.37357074	0.750430197	0.52406218
35	GO:1901566	organonitrogen compound biosynthetic process	15.3359568	-4.640779551	4.387452174	6.569682605	-3.97963099	0.760388544	0.55409463
36	GO:0016052	carbohydrate catabolic process	1.344206391	-1.373598956	-6.023383389	5.512438902	-2.597427925	0.898102019	0.57224934
37	GO:0019693	ribose phosphate metabolic process	2.92249384	-3.143120047	-4.635401424	5.84972583	-2.306098633	0.748797652	0.58053335
38	GO:0010035	response to inorganic substance	0.306269959	4.198430322	5.351346797	4.87008187	-1.502185499	0.772016629	0.58888319
39	GO:0009132	nucleoside diphosphate metabolic process	0.690020302	-5.013064596	-1.843618949	5.222836081	-2.788803167	0.699967872	0.59968967
40	GO:1901701	cellular response to oxygen-containing compound	0.400438553	4.347999627	4.805407523	4.986511975	-1.502185499	0.700908929	0.60361767
41	GO:0010106	cellular response to iron ion starvation	0.001705998	5.013665089	1.731897647	2.617000341	-1.502185499	0.709079225	0.61323807
42	GO:0044249	cellular biosynthetic process	22.87469451	-5.701694903	4.515952035	6.743326993	-3.896054232	0.783750819	0.63246679
43	GO:0009636	response to toxic substance	1.059618712	4.503341164	5.006136705	5.409122924	-1.502185499	0.748820072	0.66391396
44	GO:0034599	cellular response to oxidative stress	0.208676977	4.692855697	4.387080636	4.703454745	-1.502185499	0.681662283	0.66607993
45	GO:0006412	translation	5.346943753	-5.245337912	3.157865838	6.112077563	-5.936926355	0.670655414	0.67137328
46	GO:0062197	cellular response to chemical stress	0.231916548	4.506051887	4.308567077	4.749311086	-1.502185499	0.720921196	0.67214983
47	GO:0006090	pyruvate metabolic process	0.818337765	-5.733964056	-3.853120648	5.296906398	-2.598280076	0.816034824	0.69337288

Table S3. Metabolic process GO terms of 20 h encysting *Entamoeba invadens* circRNAs

	term_ID	description	frequency	plot_X	plot_Y	log_size	Log_pvalue	uniqueness	dispensability
1	GO:0006090	pyruvate metabolic process	0.818337765	-6.171301603	5.747187447	5.296906398	-2.076582595	0.781705906	0
2	GO:0008152	metabolic process	62.77472966	5.534082623	-0.391117381	7.181756492	-1.741871643	1	0
3	GO:0010038	response to metal ion	0.127817643	3.356084459	0.072522888	4.490576452	-2.028122311	0.973300173	0
4	GO:0048522	positive regulation of cellular process	1.943965783	2.809831687	5.35637115	5.672661151	-2.535232553	0.724096206	0
5	GO:0071103	DNA conformation change	1.133819285	4.872915312	-2.644003726	5.438517034	-1.875192689	0.951566557	0.01119524
6	GO:0071840	cellular component organization or biogenesis	9.17406249	5.450350694	1.225247703	6.346533502	-1.398425839	0.981618327	0.01513465
7	GO:0006665	sphingolipid metabolic process	0.162924845	-1.688786095	7.319411915	4.59596994	-2.028122311	0.792447918	0.057
8	GO:0006266	DNA ligation	0.104487195	-8.413896524	-0.174715582	4.403051853	-2.028122311	0.81180184	0.06375612
9	GO:0006091	generation of precursor metabolites and energy	3.310573204	-4.689837411	-7.38478826	5.903875349	-1.680261719	0.899020491	0.07789929
10	GO:1901360	organic cyclic compound metabolic process	22.66688499	-0.157642272	-4.377076406	6.739363532	-3.577445447	0.883715595	0.08360293
11	GO:0022900	electron transport chain	1.716274987	1.646743454	-6.378397195	5.61855953	-1.729097486	0.907392954	0.08561969
12	GO:0044281	small molecule metabolic process	15.96586237	2.576246692	-5.589501856	6.587164106	-1.96480618	0.920345114	0.10444611
13	GO:0009058	biosynthetic process	24.55552771	-0.258587601	-7.179377452	6.774120957	-2.184940695	0.912700638	0.12001212
14	GO:1901575	organic substance catabolic process	5.550440775	3.621884707	-4.347274342	6.128299408	-1.316248047	0.858920453	0.12018019
15	GO:1901135	carbohydrate derivative metabolic process	7.132429372	2.346632263	-3.006398622	6.237209343	-1.993838868	0.905789379	0.12748679
16	GO:0034641	cellular nitrogen compound metabolic process	26.56565976	-3.387879512	-3.48162257	6.808292286	-6.427747338	0.828719376	0.13517013
17	GO:0010467	gene expression	11.65274909	-7.663780113	-4.478549013	6.450400161	-3.230913867	0.873343264	0.15350589
18	GO:0044237	cellular metabolic process	51.61983861	-1.762729985	-6.356506297	7.096788289	-2.116329592	0.893646655	0.17447766
19	GO:0051438	regulation of ubiquitin-protein transferase activity	0.033735795	3.821567284	3.662932353	3.912115729	-1.729097486	0.86681729	0.18822471
20	GO:0043603	cellular amide metabolic process	7.027632371	-5.013242072	-0.185309844	6.230780902	-3.599022715	0.777490527	0.21274344
21	GO:0046483	heterocycle metabolic process	21.90910395	-3.253247532	-5.405179069	6.72459631	-3.577445447	0.861860125	0.2249937
22	GO:0006725	cellular aromatic compound metabolic process	21.92918763	-3.982296249	-5.641058517	6.724994237	-3.005252553	0.861834692	0.22506621
23	GO:1903320	regulation of protein modification by small protein conjugation or removal	0.073634661	1.722403952	6.23677363	4.251078264	-1.729097486	0.818060009	0.23015448
24	GO:0006807	nitrogen compound metabolic process	45.78921766	-1.143497086	-6.206501313	7.044734875	-1.51409674	0.89829902	0.24033434
25	GO:0009212	pyrimidine deoxyribonucleoside triphosphate biosynthetic process	0.084783542	-6.508187832	2.206300882	4.312304325	-2.028122311	0.641945558	0.25959376
26	GO:1901566	organonitrogen compound biosynthetic process	15.3359568	-4.070941503	-1.338234761	6.569682605	-3.409130681	0.748602707	0.2646145
27	GO:0032784	regulation of DNA-templated transcription, elongation	0.149652761	1.983162515	5.583031506	4.559068334	-1.555008513	0.852236548	0.26625219
28	GO:0048518	positive regulation of biological process	2.111108119	2.987675645	4.678086548	5.708482938	-2.535232553	0.869396129	0.30948388
29	GO:0006396	RNA processing	3.831075991	-6.818128646	-0.879792241	5.967292847	-1.41035889	0.736158695	0.33621184
30	GO:0051103	DNA ligation involved in DNA repair	0.008273882	-2.041141204	0.701178223	3.301897717	-2.028122311	0.833408601	0.34133344
31	GO:0019692	deoxyribose phosphate metabolic process	0.194516783	-3.407802699	6.832533218	4.672937913	-1.555008513	0.781104198	0.38225614
32	GO:0080090	regulation of primary metabolic process	12.49225279	2.589174178	5.070756936	6.48061252	-1.373057804	0.784195216	0.40997633
33	GO:0009084	glutamine family amino acid biosynthetic process	0.621231005	-6.568131733	3.755398264	5.177227623	-2.028122311	0.719312198	0.44165911
34	GO:0071897	DNA biosynthetic process	0.696931038	-6.861885601	-0.247622137	5.227163993	-1.729097486	0.714347761	0.46925544
35	GO:0009262	deoxyribonucleotide metabolic process	0.269522851	-5.555552825	3.243327111	4.81457386	-1.432069175	0.670849565	0.47142747
36	GO:0006139	nucleobase-containing compound metabolic process	19.00190213	-4.977444612	-0.931901053	6.662768783	-3.023340725	0.711476292	0.48170302
37	GO:0030488	tRNA methylation	0.274475614	-7.976689733	-0.629248007	4.822481914	-1.432069175	0.798421074	0.50314279
38	GO:0009263	deoxyribonucleotide biosynthetic process	0.191042826	-5.679384788	2.319921974	4.665111737	-1.555008513	0.613542407	0.50873768
39	GO:0055086	nucleobase-containing small molecule metabolic process	5.183027529	-6.031697989	1.508081658	6.098555474	-1.796211743	0.685754726	0.51321356
40	GO:0010035	response to inorganic substance	0.306269959	3.13498613	-0.102130442	4.87008187	-1.729097486	0.978532879	0.51945258
41	GO:0032787	monocarboxylic acid metabolic process	2.785819879	-6.290136404	5.070020698	5.828925289	-1.646640695	0.751079347	0.52068716
42	GO:0009059	macromolecule biosynthetic process	11.33841591	-7.094527827	-2.75116058	6.438524154	-1.930032196	0.786080853	0.54872166
43	GO:0051338	regulation of transferase activity	0.282501652	3.551294449	4.016062799	4.834998954	-1.337155675	0.864969862	0.55388264
44	GO:0044271	cellular nitrogen compound biosynthetic process	14.24186289	-5.504929714	-0.918136128	6.537538538	-3.066180489	0.702639033	0.55409463
45	GO:0009141	nucleoside triphosphate metabolic process	0.798559758	-6.110944651	2.847009406	5.286281279	-1.432069175	0.657903464	0.56981163
46	GO:0006082	organic acid metabolic process	9.351023607	-5.705149917	3.835989312	6.354830957	-1.577358116	0.730719605	0.57859047
47	GO:0006412	translation	5.346943753	-6.041617302	-0.219883528	6.112077563	-3.046455971	0.658766982	0.61354827
48	GO:0006221	pyrimidine nucleotide biosynthetic process	0.629537933	-5.922628282	2.018141995	5.182996366	-1.337155675	0.606343862	0.61864849
49	GO:0032968	positive regulation of transcription elongation from RNA polymerase II promoter	0.031149948	2.365199552	5.912719224	3.877486528	-2.028122311	0.75294933	0.62865619
50	GO:0044249	cellular biosynthetic process	22.87469451	-4.76175185	-3.058815802	6.743326993	-2.358063503	0.763231479	0.63246679
51	GO:0090304	nucleic acid metabolic process	13.86055794	-5.834396063	-1.195038333	6.525752454	-1.452082915	0.699967117	0.65202597
52	GO:0030261	chromosome condensation	0.086621723	4.929984891	-2.442106475	4.32161914	-1.432069175	0.951681973	0.65556898
53	GO:0001573	ganglioside metabolic process	0.006588538	-2.710509744	5.332363685	3.203032887	-2.028122311	0.729052557	0.65574368
54	GO:0009064	glutamine family amino acid metabolic process	1.331140844	-5.801436691	4.66109179	5.508196969	-1.337155675	0.739156431	0.67985027
